# Impact of SGLT-2 Inhibition on Cardiometabolic Abnormalities in a Rat Model of Polycystic Ovary Syndrome

**DOI:** 10.3390/ijms22052576

**Published:** 2021-03-04

**Authors:** Jacob E. Pruett, Edgar D. Torres Fernandez, Steven J. Everman, Ruth M. Vinson, Kacey Davenport, Madelyn K. Logan, Stephanie A. Ye, Damian G. Romero, Licy L. Yanes Cardozo

**Affiliations:** 1 Department of Cell and Molecular Biology, University of Mississippi Medical Center, Jackson, MS 39216, USA; jepruett@umc.edu (J.E.P.); edgar.torres.fernandez@ascension.org (E.D.T.F.); severman@umc.edu (S.J.E.); rvinson@umc.edu (R.M.V.); kdavenport@umc.edu (K.D.); mdavis8@umc.edu (M.K.L.); sye@umc.edu (S.A.Y.); dromero@umc.edu (D.G.R.); 2 Mississippi Center of Excellence in Perinatal Research, University of Mississippi Medical Center, Jackson, MS 39216, USA; 3 Women’s Health Research Center, University of Mississippi Medical Center, Jackson, MS 39216, USA; 4 Cardio Renal Research Center, University of Mississippi Medical Center, Jackson, MS 39216, USA; 5 Division of Endocrinology, Department of Medicine, University of Mississippi Medical Center, Jackson, MS 39216, USA

**Keywords:** polycystic ovary syndrome, androgens, obesity, renin-angiotensin system, blood pressure, sodium glucose cotransporter-2

## Abstract

Polycystic ovary syndrome (PCOS) is the most common endocrine disorder in reproductive-age women. PCOS is characterized by hyperandrogenism and ovulatory dysfunction. Women with PCOS have a high prevalence of obesity, insulin resistance (IR), increased blood pressure (BP), and activation of the renin angiotensin system (RAS). Effective evidence-based therapeutics to ameliorate the cardiometabolic complications in PCOS are lacking. The sodium-glucose cotransporter-2 (SGLT2) inhibitor Empagliflozin (EMPA) reduces BP and hyperglycemia in type 2 diabetes mellitus. We hypothesized that hyperandrogenemia upregulates renal SGLT2 expression and that EMPA ameliorates cardiometabolic complications in a hyperandrogenemic PCOS model. Four-week-old female Sprague Dawley rats were treated with dihydrotestosterone (DHT) for 90 days, and EMPA was co-administered for the last three weeks. DHT upregulated renal SGLT2, SGLT4, and GLUT2, but downregulated SGLT3 mRNA expression. EMPA decreased DHT-mediated increases in fat mass, plasma leptin, and BP, but failed to decrease plasma insulin, HbA1c, or albuminuria. EMPA decreased DHT-mediated increase in renal angiotensin converting enzyme (ACE), angiotensin converting enzyme 2 (ACE2), and angiotensin II type 1 receptor (AGT1R) mRNA and protein expression. In summary, SGLT2 inhibition proved beneficial in adiposity and BP reduction in a hyperandrogenemic PCOS model; however, additional therapies may be needed to improve IR and renal injury.

## 1. Introduction

Polycystic ovary syndrome (PCOS) is the most common endocrine disorder in reproductive-age women, affecting between 5–20% of women in this demographic [1,2,3]. PCOS is characterized by androgen excess, ovulatory dysfunction, and polycystic appearance of the ovaries [4]. The etiology of PCOS is currently unknown. The sources of excess androgens in PCOS are the ovaries, the adrenals, and peripheral tissues, such as the adipose tissue, which convert pro-androgens into active metabolites [5,6,7,8].

PCOS is associated with cardiometabolic risk factors such as obesity, insulin resistance (IR)/type 2 diabetes mellitus (T2DM), hyperleptinemia, increased blood pressure (BP), and increased urinary albumin to creatinine ratio [4,9,10,11,12]. Patients with PCOS currently have very few effective evidence-based pharmacological agents for treating their cardiometabolic complications [13,14].

Sodium-glucose cotransporter-2 (SGLT2) reabsorbs both glucose and sodium in the proximal renal tubule [15], and it is responsible for about 90% of the glucose reabsorption in the nephron [16]. SGLT2 inhibitors are therapeutic agents to treat hyperglycemia in patients with T2DM. These agents were also shown to be promising treatment options for patients with heart failure and kidney disease, even in the absence of diabetes [17]. Empagliflozin (EMPA) was the first anti-diabetic agent shown to reduce cardiovascular mortality in patients with T2DM in the EMPA-REG trial, showing a 38% relative risk reduction in cardiovascular death [18]. A recent 13-week small clinical trial in women with PCOS showed that SGLT2 inhibitors decreased body weight (BW), body mass index, and fat mass. However, EMPA did not modify IR, lipid profile, androgenemia, or BP [14]. Those results are in contrast with the proven BP and HbA1c lowering effects consistently shown in humans [16,19], and rodent models of types 1 [20] and 2 [21,22] diabetes mellitus. Thereby, whether and how SGLT2 inhibition ameliorates the cardiometabolic complications in PCOS is not completely understood.

Androgens have been shown to modulate SGLT2 expression in the kidneys of male rodents [15], where castration increased renal SGLT2 protein expression and testosterone restored the levels of SGLT2 in castrated male rats [15]. Hyperandrogenemia is a cardinal feature in women with PCOS. However, whether androgens modulate renal SGLT2 in PCOS is unclear.

Women with PCOS were shown to have an upregulation of the renin-angiotensin system (RAS) with elevated renin in the blood [23], and blockade of the RAS was effective in decreasing their elevated BP [24]. We previously showed that hyperandrogenemic female rats have an upregulation of intrarenal angiotensinogen and angiotensin-converting enzyme (ACE) mRNA [25]. Interactions between SGLT2 and the RAS were reported in preclinical studies [26,27]. SGLT2 co-localizes with multiple components of the intrarenal RAS in proximal tubular cells [28]. Whether SGLT2 inhibition will impact the intrarenal RAS and lower BP in PCOS remains unknown. 

We showed that the hyperandrogenemic rat model of PCOS recapitulates several cardiometabolic features observed in women with PCOS, including increased body mass index (BMI), IR, BP, hyperleptinemia, albuminuria, and activation of the intrarenal RAS [25,29,30]. In this study, we hypothesized that androgens upregulate the expression of renal SGLT2 and that SGLT2 inhibition ameliorates the cardiometabolic complications in an experimental model of PCOS.

## 2. Results

### 2.1. Effect of Hyperandrogenemia on Renal Glucose Transporter mRNA Expression

**Figure 1:**
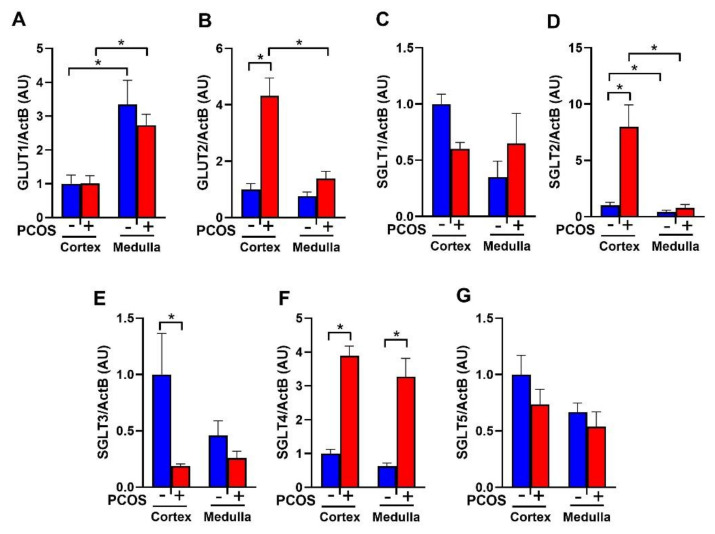
Renal glucose transporters mRNA expression in polycystic Ovary Syndrome (PCOS). Renal cortical and medullar mRNA expression of (**A**) GLUT1, (**B**) GLUT2, (**C**) SGLT1, (**D**) SGLT2, (**E**) SGLT3, (**F**) SGLT4, and (**G**) SGLT5 after 90 days of dihydrotestosterone (DHT) treatment. Data are expressed as mean ± SEM. Data were analyzed by two-way ANOVA followed by Tukey post-hoc tests. Significant interaction was only observed for SGLT2 and GLUT2. * *p* < 0.05. n = 6–8 per group. PCOS: Polycystic Ovary Syndrome.

PCOS rats at 16 weeks of age had no change in renal GLUT1 expression compared to controls (Figure 1A). In contrast, PCOS rats had a significant increase in cortical GLUT2 expression (4.32 ± 1.81 vs. 1.00 ± 0.21, *p* < 0.0001) compared to controls (Figure 1B). PCOS rats had no change in renal SGLT1 mRNA expression compared to controls (Figure 1C). There was a ~7-fold increase in SGLT2 mRNA in PCOS renal cortex compared to controls (7.17 ± 1.76 vs. 1.00 ± 0.19, *p* < 0.001) (Figure 1D). SGLT3 was downregulated in the cortex of PCOS rats compared to controls (0.19 ± 0.02 vs. 1.00 ± 0.36, *p* < 0.05) (Figure 1E). SGLT4 was upregulated in PCOS in both the cortex (3.89 ± 0.29 vs. 1.00 ± 0.12, *p* < 0.0001) and the medulla (3.27 ± 0.54 vs. 0.62 ± 0.09, *p* < 0.0001) (Figure 1F). There were no changes in SGLT5 expression between PCOS rats and controls (Figure 1G).

### 2.2. Effect of EMPA on Body Weight, Food Intake, and Fluid Intake in PCOS Model

**Figure 2:**
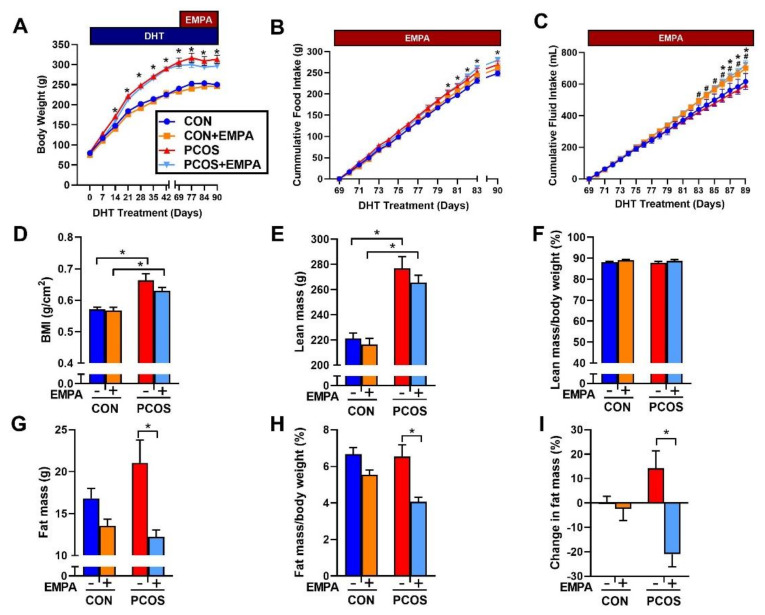
Effect of Empagliflozin (EMPA) on anthropomorphic measures in PCOS. Effect of EMPA on (**A**) BW, (**B**) cumulative food intake, (**C**) cumulative fluid intake, (**D**) body mass index (BMI), (**E**) total lean mass, (**F**) lean mass corrected by body weight (BW), (**G**) total fat mass, (**H**) fat mass corrected by BW, and (**I**) percent change in fat mass before and after three weeks of EMPA treatment. Data are expressed as mean ± SEM. Data were analyzed by two-way repeated measures ANOVA followed by Tukey post-hoc tests. Significant interaction was only observed for BW and percent change in fat mass. * *p* < 0.05 vs. untreated control rats; ^#^ *p* < 0.05 vs. untreated PCOS rats. n = 7–10 per group. CON: Control, CON+EMPA: Control+Empagliflozin, PCOS: Polycystic Ovary Syndrome, PCOS+EMPA: Polycystic Ovary Syndrome+Empagliflozin.

PCOS rats at 16 weeks of age increased BW compared to controls (313.3 ± 10.3 vs. 250.2 ± 5.8 g, *p* < 0.0001) (Figure 2A). EMPA treatment did not affect BW in either group. PCOS rats had increased cumulative food intake when compared with controls (269.3 ± 8.3 vs. 248.6 ± 6.3 g, *p* < 0.05), and EMPA did not affect cumulative food intake among either controls or PCOS rats (Figure 2B). EMPA treatment increased cumulative fluid intake in both groups, PCOS (725 ± 25 vs. 593 ± 25 mL, *p* < 0.0001) and controls (701 ± 32 vs. 617 ± 50 mL, *p* < 0.05) (Figure 2C).

### 2.3. Effect of EMPA on Body Composition in PCOS Model

PCOS rats at 16 weeks of age had a higher BMI than controls (0.664 ± 0.020 vs. 0.571 ± 0.007 g/cm^2^, *p* < 0.0001). EMPA did not modify BMI in either PCOS (0.630 ± 0.010 vs. 0.664 ± 0.020 g/cm^2^, *p* = 0.2958) or controls (0.568 ± 0.010 vs. 0.571 ± 0.007 g/cm^2^, *p* = 0.9977) (Figure 2D). As shown in Figure 2E and 2F, PCOS rats at 16 weeks had a higher total lean mass than controls (277.0 ± 9.3 vs. 221.3 ± 4.2 g, *p* < 0.0001), and while PCOS rats had higher fat mass compared to controls, it did not reach statistical significance (21.1 ± 2.7 vs. 16.8 ± 1.2 g, *p* = 0.253) (Figure 2G). As shown in Figure 2F,H, when lean mass and fat mass were corrected by BW, there were no differences between PCOS rats and controls. EMPA did lower total fat mass (12.2 ± 0.8 vs. 21.1 ± 2.7 g, *p* < 0.01) (Figure 2G) and fat mass corrected by BW (4.1 ± 0.2 vs. 6.5 ± 0.6%, *p* < 0.001) in PCOS rats (Figure 2H). After three weeks of EMPA treatment, fat mass was significantly reduced in PCOS model when corrected by BW while untreated PCOS rats had increased fat mass (−21.0 ± 5.1 vs. 14.2 ± 7.2%, *p* < 0.001) as shown in Figure 2I. EMPA had no impact on either total lean mass or lean mass corrected by BW in either controls or PCOS rats (Figure 2E,F).

### 2.4. Effect of EMPA on Insulin Sensitivity, HbA1c, Fasting Lipid Profile, and Leptin in PCOS Model

**Figure 3:**
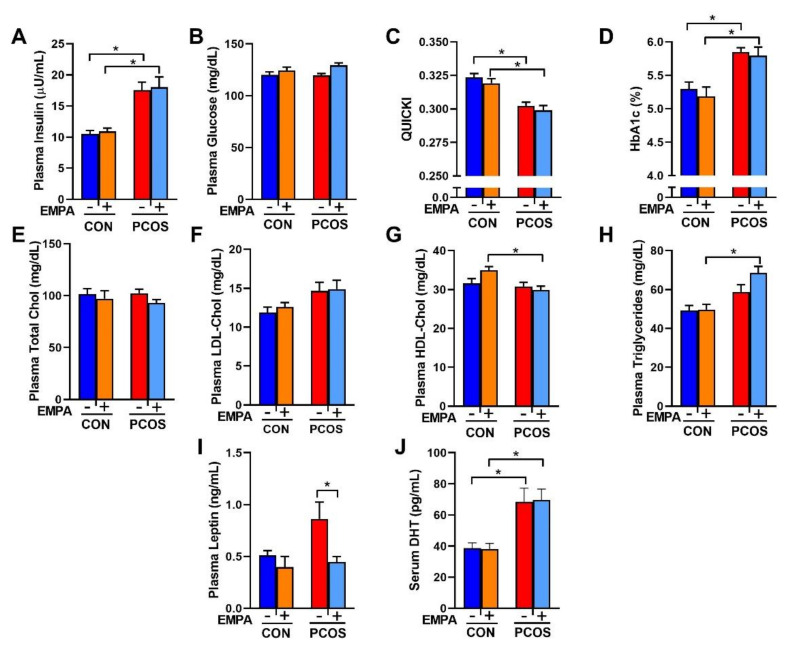
Effect of EMPA on metabolic parameters in PCOS. (**A**–**C**) Effect of EMPA on (**A**) fasting plasma insulin, (**B**) fasting plasma glucose, and (**C**) insulin sensitivity (QUICKI). (**D**–**H**) Effect of EMPA on (**D**) hemoglobin A1c (HbA1c), (**E**) fasting plasma total cholesterol, (**F**) fasting plasma low-density lipoprotein cholesterol (LDL-Chol), (**G**) fasting plasma high-density lipoprotein cholesterol (HDL-Chol), (**H**) fasting plasma triglycerides, (**I**) plasma leptin, and (**J**) serum DHT. Data are expressed as mean ± SEM. Data were analyzed by two-way ANOVA followed by Tukey post-hoc tests. Significant interaction was observed only for HDL-Chol. * *p* < 0.05. n = 7–10 per group.

As shown in Figure 3A, PCOS rats at 16 weeks of age had higher fasting insulin than control rats (17.6 ± 1.3 vs. 10.5 ± 0.5 µU/mL, *p* < 0.01). There were no differences in fasting plasma glucose between PCOS rats and controls (119 ± 2 vs. 120 ± 3 mg/dL, *p* = 0.997) as shown in Figure 3B. PCOS rats had decreased insulin sensitivity compared to controls when assessed by QUICKI (0.302 ± 0.003 vs. 0.324 ± 0.003, *p* < 0.001), as shown in Figure 3C. EMPA had no impact on fasting insulin, fasting plasma glucose, or insulin sensitivity in either PCOS or control rats. PCOS rats also had increased HbA1c compared to controls. After 3 weeks of EMPA treatment, EMPA had no impact on HbA1c in either PCOS rats (5.794 ± 0.129 vs. 5.850 ± 0.063%, *p* = 0.986) or control rats (5.187 ± 0.137 vs. 5.295 ± 0.105%, *p* = 0.912) (Figure 3D).

There were no differences between PCOS rats and controls in fasting total cholesterol, LDL cholesterol (LDL-Chol), HDL cholesterol (HDL-Chol), or triglycerides, as illustrated in Figure 3E–H. However, when compared to EMPA-treated controls, EMPA-treated PCOS rats had lower fasting HDL cholesterol (29.9 ± 0.9 vs. 35.0 ± 0.9 mg/dL, *p* < 0.01) and higher fasting triglycerides (68.6 ± 2.3 vs. 49.7 ± 2.8 mg/dL, *p* < 0.001). Plasma leptin was increased in PCOS rats compared to controls, although it did not reach statistical significance (0.86 ± 0.16 vs. 0.51 ± 0.05 ng/mL, *p* = 0.0949), as shown in Figure 3I. EMPA decreased plasma leptin in PCOS rats to levels similar to the control group (0.45 ± 0.05 vs. 0.86 ± 0.16 ng/mL, *p* < 0.05), and did not modify leptin levels in controls. PCOS rats had higher serum dihydrotestosterone (DHT) levels (68.6 ± 8.7 vs. 38.7 ± 3.4 pg/mL, *p* < 0.01) compared to controls and were not affected by EMPA treatment (Figure 3J).

### 2.5. Effect of EMPA on Urinary Glucose, Ketone, and Albumin Excretion and Kidney Weight in a PCOS Model

**Figure 4:**
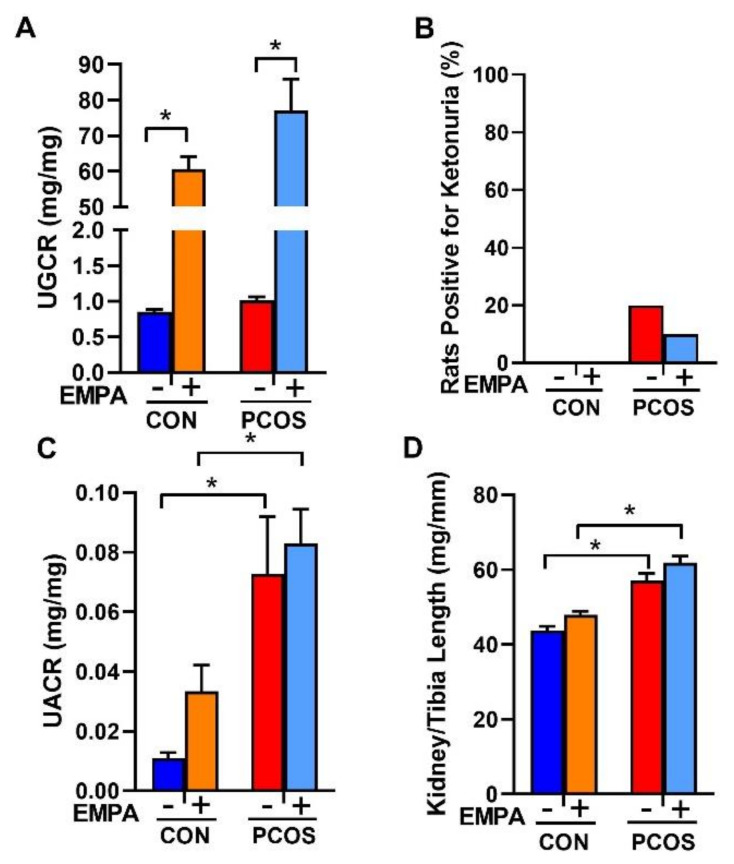
Effect of EMPA on urinary glucose, ketone, and albumin excretion on kidney hypertrophy in PCOS. Effect of EMPA on (**A**) urine glucose to creatinine ratio (UGCR), (**B**) urine ketone excretion, (**C**) urine albumin to creatinine ratio (UACR), and (**D**) kidney weight corrected by tibia length. Data are expressed as mean ± SEM. Data were analyzed by two-way ANOVA followed by Tukey post-hoc tests. No significant interactions were observed by two-way ANOVA. * *p* < 0.05. n = 7–10 per group.

As shown in Figure 4A, EMPA increased the urinary glucose to creatinine ratio in both controls (60.65 ± 3.47 vs. 0.85 ± 0.03 mg/mg, *p* < 0.0001) and PCOS rats (77.05 ± 8.88 vs. 1.01 ± 0.05 mg/mg, *p* < 0.0001). As shown in Figure 4B, no urinary ketones were detected in any of the treated or untreated controls. Only 20% of untreated PCOS rats and 10% of EMPA treated PCOS rat had a trace level of ~5 mg/dL of ketones. The rest of the PCOS rats, whether treated or untreated, had no detectable urinary ketones. As illustrated in Figure 4C, PCOS rats had a higher urinary albumin to creatinine ratio compared to controls (0.073 ± 0.019 vs. 0.011 ± 0.002 mg/mg, *p* < 0.01). EMPA treatment did not affect urinary albumin to creatinine ratio in either controls or PCOS rats. As shown in Figure 4D, at the end of the experimental period, PCOS rats compared to controls had an increased kidney weight to tibia length ratio (61.9 ± 1.7 vs. 43.7 ± 1.2 mg/mm, *p* < 0.0001). EMPA had no impact on kidney weight in either controls or PCOS rats.

### 2.6. Effect of EMPA on Blood Pressure in PCOS Model

**Figure 5:**
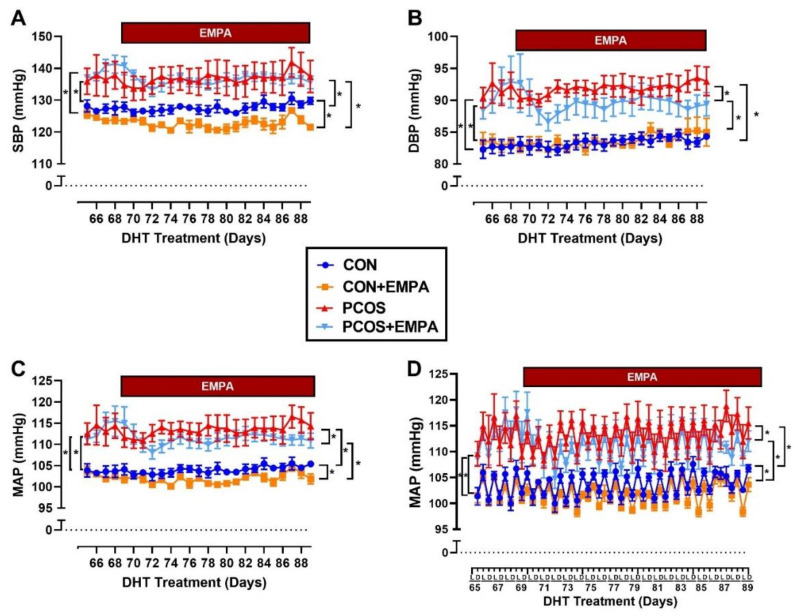
Effect of EMPA on blood pressure and heart rate in PCOS. (**A**–**C**) Effect of EMPA on (**A**) systolic BP (SBP), (**B**) diastolic BP (DBP), and (**C**) mean arterial pressure (MAP) during last 3 weeks of DHT, with 4 days of baseline measurements. (**D**) Effect of EMPA on the light (L) and dark (**D**) phases of the circadian rhythm of MAP during the last 3 weeks of DHT, with 4 days of baseline. Data are expressed as mean ± SEM. Data was analyzed by two-way repeated measures ANOVA followed by Tukey post-hoc tests, with separate analyses for both baseline and for during EMPA treatment. No significant interactions were observed by two-way repeated measures ANOVA. * *p* < 0.05. n = 5–7 per group. SBP: Systolic blood pressure, DBP: Diastolic blood pressure, MAP: Mean arterial pressure.

As shown in Figure 5A–C, PCOS rats had significantly higher SBP (systolic blood pressure) (137 ± 1 vs. 127 ± 1 mmHg, *p* < 0.0001), DBP (diastolic blood pressure) (92 ± 1 vs. 83 ± 1 mmHg, *p* < 0.0001) and MAP (mean arterial pressure) (114 ± 1 vs. 104 ± 1 mmHg, *p* < 0.0001) at baseline compared to control group. At baseline, there were no differences between treated and untreated PCOS or control rats in SBP, DBP, or MAP. After three weeks of EMPA treatment when compared to untreated PCOS rats, EMPA treated PCOS rats had unchanged SBP (136 ± 1 vs. 137 ± 1 mmHg, *p* = 0.936). However, the EMPA treated PCOS rats had a small but significant decrease in DBP (89 ± 1 vs. 92 ± 1 mmHg, *p* < 0.0001) and MAP (111 ± 1 vs. 113 ± 1 mmHg, *p* < 0.001). At the end of the experimental protocol when compared to untreated controls, EMPA treated control rats had decreased SBP (123 ± 1 vs. 128 ± 1 mmHg, *p* < 0.0001), unchanged DBP (84 ± 1 vs. 83 ± 1 mmHg, *p* = 0.958), and decreased MAP (102 ± 1 vs. 104 ± 1 mmHg, *p* < 0.001). In addition, PCOS rats had a higher left ventricle mass to tibia length ratio (19.8 ± 0.7 vs. 16.7 ± 0.5 mg/mm, *p* < 0.01) compared to controls. EMPA had no impact on the left ventricle mass to tibia length ratio in either PCOS or control rats (Control: 16.7 ± 0.6, Control+EMPA: 15.2 ± 0.3, PCOS: 19.8 ± 0.8, and PCOS+EMPA: 20.3 ± 0.6 mg/mm).

### 2.7. Effect of EMPA on Circadian Rhythm of Blood Pressure in PCOS Model

As shown in Figure 5D at baseline, PCOS rats had higher MAP than controls in both light (111 ± 1 vs. 101 ± 1 mmHg, *p* < 0.0001) and dark (116 ± 1 vs. 106 ± 1 mmHg, *p* < 0.0001) phases. At baseline, there were no differences in MAP between PCOS treated and untreated rats in either the light (111 ± 1 vs. 111 ± 1 mmHg, *p* = 0.989) or dark (116 ± 1 vs. 116 ± 1 mmHg, *p* = 0.999) phase. There were also no differences at baseline in MAP between treated and untreated control rats in either the light or dark phase. After three weeks of EMPA treatment when compared to untreated PCOS rats, EMPA treated PCOS rats had reduced MAP in both the light (108 ± 1 vs. 111 ± 1 mmHg, *p* < 0.0001) and the dark (114 ± 1 vs. 116 ± 1 mmHg, *p* < 0.01) phase. Similarly, after three weeks of EMPA treatment when compared to untreated control rats, EMPA treated control rats had reduced MAP in both the light (100 ± 1 vs. 102 ± 1 mmHg, *p* < 0.01) and the dark (103 ± 1 vs. 106 ± 1 mmHg, *p* < 0.0001) phase.

### 2.8. Effect of EMPA on the mRNA Expression of the Intrarenal Renin-Angiotensin System in PCOS Model

**Figure 6:**
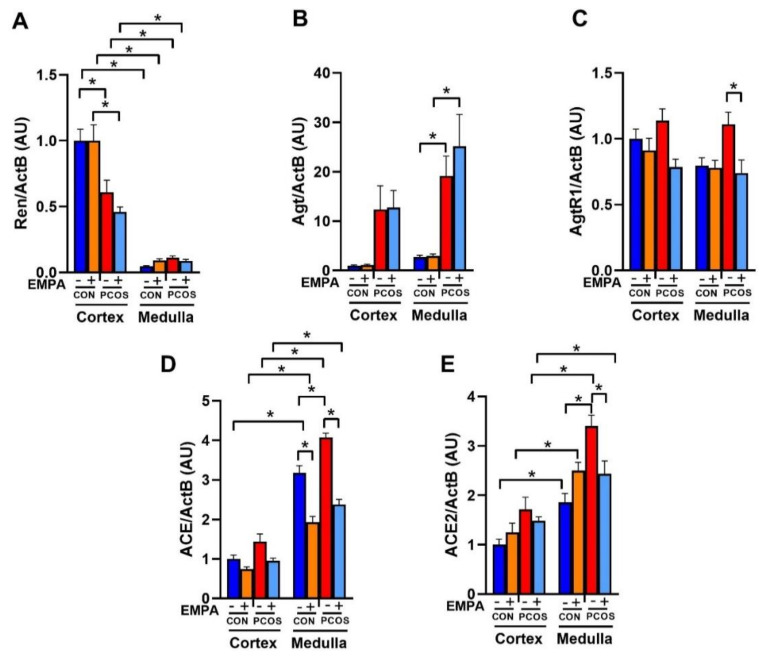
Effect of EMPA on mRNA expression of the intrarenal renin-angiotensin system in PCOS. Effect of EMPA on the renal cortical and medullar mRNA expression of (**A**) Ren (Renin), (**B**) Agt (Angiotensinogen), (**C**) AgtR1 (AT1R), and (**D**) ACE (angiotensin-converting enzyme), and (**E**) ACE2 (angiotensin-converting enzyme 2) after 3 weeks of EMPA treatment. Data are expressed as mean ± SEM. Data were analyzed by three-way ANOVA followed by Tukey post-hoc tests. Significant interaction was observed only for ACE2. * *p* < 0.05. n = 7–10 per group.

As shown in Figure 6A, PCOS rats had a downregulation of renin mRNA compared to controls in the renal cortex (0.61 ± 0.09 vs. 1.00 ± 0.09, *p* < 0.01). As shown in Figure 6B, in PCOS rats compared to controls, angiotensinogen mRNA was significantly upregulated in the renal medulla (19.17 ± 4.00 vs. 2.71 ± 0.40, *p* < 0.05) and elevated in the cortex without reaching significance by 3-way ANOVA (12.35 ± 4.80 vs. 1.00 ± 0.16, *p* = 0.298). EMPA treatment in PCOS rats did not reverse these alterations in renal angiotensinogen or renin mRNA expression compared to untreated PCOS rats.

As shown in Figure 6C, angiotensin II type 1 receptor (AT1R) mRNA expression was unchanged between PCOS rats and controls in both the renal cortex and medulla. EMPA treatment in PCOS rats downregulated AT1R mRNA expression in the medulla (0.74 ± 0.10 vs. 1.11 ± 0.09, *p* < 0.05) and cortex (0.79 ± 0.06 vs. 1.14 ± 0.09, *p* = 0.055). In control rats, EMPA had no effect on AT1R expression in either the cortex or medulla.

As shown in Figure 6D, angiotensin-converting enzyme (ACE) mRNA was significantly upregulated in PCOS rats compared to controls in the renal medulla (4.07 ± 0.11 vs. 3.18 ± 0.18, *p* < 0.001), and elevated in the renal cortex without reaching significance (1.44 ± 0.19 vs. 1.00 ± 0.10, *p* = 0.271). EMPA treatment in PCOS rats decreased ACE mRNA expression compared to untreated PCOS rats in the renal medulla (2.38 ± 0.13 vs. 4.07 ± 0.11, *p* < 0.0001), and in the renal cortex (0.96 ± 0.07 vs. 1.44 ± 0.19, *p* = 0.194). EMPA treatment also decreased ACE mRNA expression in the renal medulla of treated controls versus untreated controls (1.93 ± 0.15 vs. 3.18 ± 0.18, *p* < 0.0001), without affecting cortical ACE mRNA expression. As shown in Figure 6E, angiotensin-converting enzyme 2 (ACE2) mRNA was significantly upregulated in PCOS rats compared to controls in the renal medulla (3.40 ± 0.22 vs. 1.86 ± 0.18, *p* < 0.0001), and elevated without reaching significance in the cortex (1.72 ± 0.24 vs. 1.00 ± 0.11, *p* = 0.162). EMPA treatment decreased medullar ACE2 mRNA expression in PCOS compared to untreated PCOS (2.43 ± 0.26 vs. 3.40 ± 0.22, *p* < 0.05), though it did not affect cortical expression. EMPA did not affect renal ACE2 mRNA expression between treated and untreated controls.

### 2.9. Effect of EMPA on Intrarenal ACE and ACE2 Protein Expression and Activity in PCOS Model

**Figure 7:**
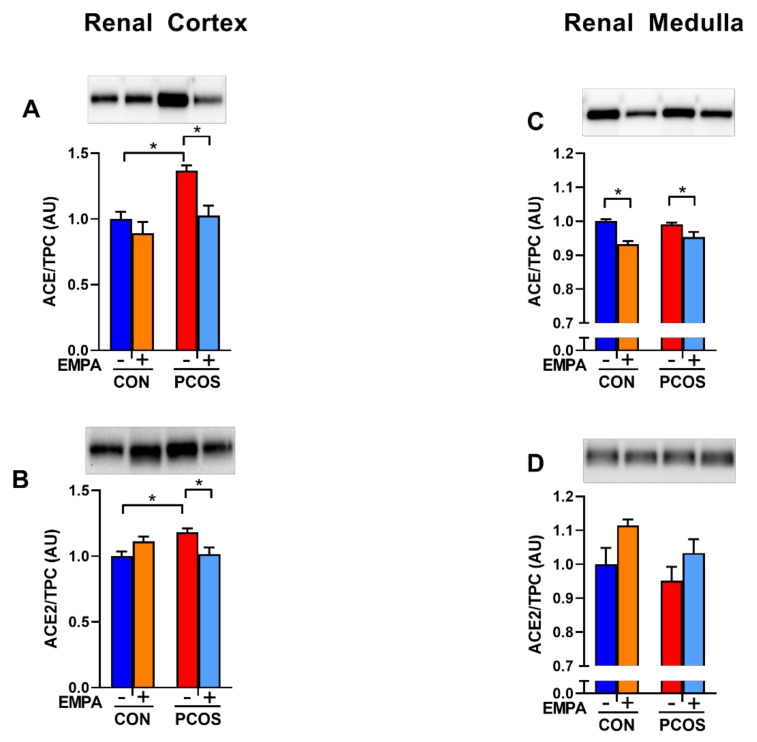
Effect of EMPA on renal ACE and ACE2 protein expression in PCOS. Effect of EMPA on renal (**A**) cortical Angiotensin-Converting Enzyme (ACE), (**B**) cortical angiotensin-converting enzyme 2 (ACE2), (**C**) medullar ACE, and (**D**) medullar ACE2 protein expression after 3 weeks of EMPA treatment. Data normalized by total protein content (TPC). Data are expressed as mean ± SEM. Corresponding stain free images TPC quantification in Supplementary Figure S1. Data were analyzed by two-way ANOVA followed by Tukey post-hoc tests to avoid comparing between gels. Significant interaction was observed only for cortical ACE2. * *p* < 0.05. n = 3–5 per group.

As shown in Figure 7A, renal cortical ACE protein expression was significantly upregulated in PCOS compared to controls (1.37 ± 0.04 vs. 1.00 ± 0.06, *p* < 0.05). EMPA downregulated cortical ACE in PCOS rats (1.03 ± 0.08 vs. 1.37 ± 0.04, *p* < 0.05), but not in controls. As illustrated in Figure 7B, renal cortical ACE2 protein expression was upregulated in PCOS compared to controls (1.18 ± 0.03 vs. 1.00 ± 0.04, *p* < 0.05). EMPA downregulated cortical ACE2 in PCOS rats (1.02 ± 0.05 vs. 1.18 ± 0.03, *p* < 0.05), but not in controls.

As demonstrated in Figure 7C,D, in the renal medulla, there was no difference between PCOS rats and controls in ACE and ACE2 protein expression. EMPA decreased medullar ACE protein in both PCOS rats (0.954 ± 0.015 vs. 0.991 ± 0.005, *p* < 0.05) and controls (0.933 ± 0.009 vs. 1.000 ± 0.006, *p* < 0.01). EMPA had no impact on medullar ACE2 protein expression in either PCOS rats or controls.

**Figure 8:**
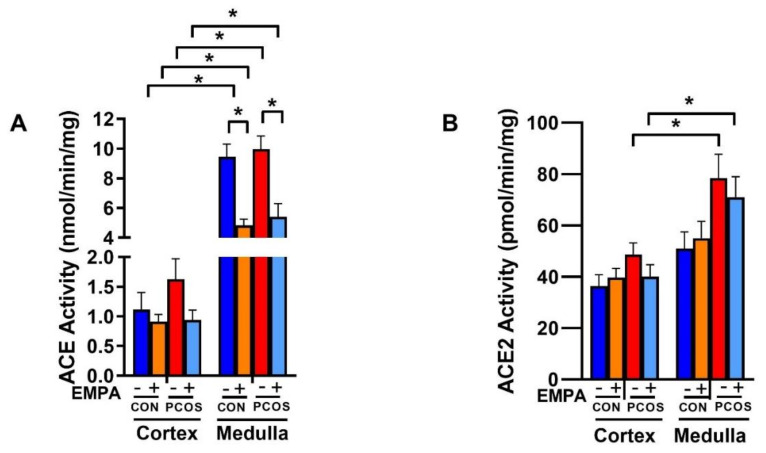
Effect of EMPA on ACE and ACE2 enzymatic activity in PCOS. Effect of EMPA on the renal cortical and medullar (**A**) angiotensin-converting enzyme (ACE) and (**B**) angiotensin-converting enzyme 2 (ACE2) enzymatic activity after 3 weeks of EMPA treatment. Data are expressed as mean ± SEM. Data were analyzed by three-way ANOVA followed by Tukey post-hoc tests. No significant interaction was observed by three-way ANOVA. * *p* < 0.05. n = 7–10 per group.

As shown in Figure 8A, intrarenal ACE activity was significantly higher in the medulla in both PCOS rats (9.965 ± 0.890 vs. 1.625 ± 0.345 nmol/min.mg protein, *p* < 0.0001) and controls (9.441 ± 0.856 vs. 1.115 ± 0.287 nmol/min.mg protein, *p* < 0.0001) compared to the renal cortex. Renal cortical ACE activity showed a tendency to increase in PCOS compared to controls, but did not reach significance (1.625 ± 0.345 vs. 1.115 ± 0.287 nmol/min.mg protein, *p* = 0.999). In PCOS rats, although renal cortical ACE activity decreased with EMPA, it did not reach statistical significance (0.939 ± 0.168 vs. 1.625 ± 0.345 nmol/min.mg protein, *p* = 0.991). There was also no change in ACE activity between PCOS and control groups in the medulla (9.965 ± 0.890 vs. 9.441 ± 0.856 nmol/min.mg protein, *p* = 0.998). EMPA treatment significantly decreased renal medullary ACE activity in both control (4.828 ± 0.418 vs. 9.441 ± 0.856 nmol/min.mg protein, *p* < 0.0001) and PCOS groups (5.414 ± 0.885 vs. 9.965 ± 0.890 nmol/min.mg protein, *p* < 0.0001).

As demonstrated in Figure 8B, intrarenal ACE2 activity was significantly higher in the medulla in PCOS rats (78.468 ± 9.311 vs. 48.707 ± 4.515 pmol/min.mg protein, *p* < 0.05) compared to the PCOS cortex, and unchanged in controls. In the renal cortex and medulla, there was an increase in ACE2 activity in PCOS compared to controls that was decreased with EMPA, though neither comparison reached statistical significance.

## 3. Discussion

Women with PCOS have a high prevalence of cardiometabolic risk factors; however, effective evidence-based therapeutic agents to ameliorate those cardiometabolic risk factors are lacking. Our study shows that androgens upregulate renal SGLT2, SGLT4, and GLUT2, but downregulates SGLT3 expression in an animal experimental model of PCOS. Moreover, our study shows that inhibition of SGLT2 with EMPA decreases fat mass, plasma leptin, and BP but fails to improve IR, HbA1c, or albuminuria in an animal experimental model of PCOS. The EMPA-mediated reduction in BP in PCOS rats could be explained by an amelioration of the androgen-induced increase in intrarenal ACE expression and activity.

Renal SGLT2 expression is regulated by androgens in male rats. Sabolic et al. showed that SGLT2 protein in the kidney cortex is higher in female rats compared to male rats [15]. Furthermore, male rats that were gonadectomized showed an upregulation of SGLT2 protein compared to control male rats. However, gonadectomized female rats showed no changes in SGLT2 expression compared to control females, leading Sabolic et al. to suggest that androgens play a more critical role for the observed sex difference than estrogens [15]. Androgens cause differential effects between males and females in several cardiometabolic risk factors, such as in obesity. For instance, in humans, increased obesity and visceral adiposity is associated with hypoandrogenemia in males, while increased obesity in females is associated with hyperandrogenemia in PCOS [31]. In our study, we found that hyperandrogenemia in female rats increased SGLT2 mRNA expression in the renal cortex. Since SGLT2 co-transports glucose and sodium, activation of SGLT-2 could be one of the mechanisms by which androgens increases BP in PCOS.

In our study, we observed that SGLT2 mRNA was not the only renal glucose transporter that was regulated by androgens. While SGLT2 is responsible for the vast majority of glucose reuptake in the nephron under normal conditions, SGLT1 is responsible for reabsorbing the glucose that escapes SGLT2 [32]. Additionally, as both SGLT2 and SGLT1 transport glucose into the proximal tubule cells, Glucose Transporter-2 (GLUT2) and Glucose Transporter-1 (GLUT1) transport glucose into the interstitium and bloodstream [33]. We observed that GLUT2 mRNA was upregulated in the renal cortex of PCOS rats, similar to SGLT2 mRNA. However, SGLT1 and GLUT1 were unchanged in our study. Sabolic et al. showed that SGLT1 mRNA was downregulated in the outer stripe of the renal medulla of male rats compared to those of female rats, though there was no sex difference in mRNA expression in the renal cortex [34]. The lack of SGLT1 mRNA downregulation in the renal medulla in PCOS rats also suggested different modulation of SGLT1 by androgens in female compared to male rats. Additionally, we analyzed the mRNA expression of other renal SGLTs such as SGLT3, SGLT4, and SGLT5. Renal SGLT3 is a sodium channel that senses glucose instead of a sodium-glucose cotransporter [35], and it shares about 70% of the amino acid sequence of SGLT1 [35]. We found that SGLT3 was downregulated by hyperandrogenemia in the renal cortex. We also observed that SGLT4, which transports mannose, glucose, and fructose in the kidney and small intestine [36], was upregulated by hyperandrogenemia in the renal cortex and medulla. Interestingly, we did not observe any changes in SGLT5 mRNA expression in the kidney; SGLT5 transported mannose and fructose similarly to SGLT4, but was only expressed in the kidney [36]. The role that SGLT4 plays in the cardiometabolic complications in PCOS is unknown at present.

Our PCOS model mimics several of the cardiometabolic features observed in women with PCOS, such as increases in BP and BMI, and decreases in insulin sensitivity. BP in this PCOS model is elevated by ~10 mmHg [25,29,30]. We also previously reported that blocking the RAS with an ACE inhibitor eliminated BP differences between treated PCOS and control rats [30], implicating activation of the RAS in the elevation of BP in our model. Indeed, women with PCOS have hyperreninemia [23] and blockade of the AT1R was effective in decreasing the BP of women with PCOS [24]. In the current study, untreated PCOS rats had ~10 mmHg increase in MAP accompanied by upregulation of intrarenal angiotensinogen mRNA expression as well as upregulation of ACE mRNA and protein compared to untreated controls, suggesting that a dysregulated intrarenal RAS is at least partially responsible for the elevated BP observed in PCOS model. With EMPA treatment, although we observed a minimal effect in BP, there was also a decrease of renal ACE mRNA, protein, and enzymatic activity along with decreased AT1R mRNA in treated PCOS rats. One of the mechanisms by which SGLT2 inhibitors lower BP in humans and preclinical models is natriuresis, especially early on in the treatment [37]. Thereby the amelioration of the intrarenal RAS activity by SGLT2 inhibition observed in our study could be specific to androgen-induced increases in BP. Furthermore, this small reduction in MAP did not result in LV size changes in the PCOS rats. In women with PCOS treated with EMPA, there were no significant changes in BP [14]. Women in that study had their BP measured three times at baseline and three times after 12 weeks of EMPA treatment [14]. Our study measured BP over 24 h in freely moving, conscious rats, which allowed us to detect even a small two mmHg decrease in MAP. This relatively small decrease in MAP may be due to the lack of significant downregulation in renal angiotensinogen or renin expression in EMPA-treated PCOS rats. The conversion of angiotensinogen to angiotensin I by renin is the rate-limiting step of the RAS [38]. Woods et al. showed that decreased angiotensinogen was associated with decreased BP by SGLT2 inhibition in males in a T2DM model [27]. Therefore, it could be speculated that for SGLT2 inhibition to cause a sizeable effect in BP, changes in angiotensinogen must be present, although this hypothesis needs to be tested. Our study shows that PCOS rats have an increase in renal ACE2 protein and activity. ACE2 is the receptor for the Severe Acute Respiratory Syndrome Coronavirus 2 (SARS-CoV-2), which is the causal agent of Coronavirus Disease 2019 (COVID-19). It is possible to speculate that women with PCOS will have a higher incidence of SARS-CoV-2 infection kidney-related problems, and probably increased morbidity and mortality due to increased renal ACE2; however, this remains to be proven [39].

There were ambiguous data in the literature about the effect of SGLT2 inhibitors and the renal RAS. Woods et al. showed SGLT2 inhibition decreased renal angiotensinogen mRNA expression in a rat model of T2DM with no changes in renal ACE or AT1R mRNA expression [27]. Meanwhile, Shin et al. showed that SGLT2 inhibition decreased AT1R protein expression in the renal cortex [40]. The landmark EMPA-REG trial for patients with T2DM demonstrated that EMPA decreased cardiovascular mortality in patients with cardiovascular disease already receiving the standard of care (i.e., ACE inhibitors or ARBs) [18]. Furthermore, in Dahl salt-sensitive rats treated with streptozotocin, it was shown that while SGLT2 inhibition did not decrease MAP by itself, SGLT2 inhibition significantly enhanced the reduction of MAP when combined with an ACE inhibitor [20]. Our study confirmed our previous findings of activation of the vasoconstrictor arm of the intrarenal RAS [25]. Furthermore, SGLT2 inhibition decreased renal ACE expression and activity as well as the AT1R mRNA expression in the PCOS model, highlighting the important role of the intrarenal ACE in regulating BP in the PCOS model and as a new mechanism of the beneficial effect of the SGLT2 inhibitors.

The decrease in fat mass in the PCOS rats treated with EMPA is in line with data from women with PCOS treated with SGLT2 inhibitors [14]. Recently, a novel anti-obesity mechanism of SGLT2 inhibition was reported, where EMPA treatment decreased adipose macrophage infiltration and increased energy expenditure in a model of diet-induced obesity [41]. In our study, changes in the adipose tissue are associated with decreased plasma leptin. Leptin stimulates the sympathetic nervous system [42], and its elevation is associated with PCOS [12]. SGLT2 inhibition was shown to reduce the activity of the sympathetic nervous system [43], therefore, the decrease in leptin may have led to decreased activation of the sympathetic nervous system, which may partially explain the decrease in BP we observed. Renal sympathetic nervous system activation stimulates the RAS [42], so decreased leptin in PCOS-treated rats may partially account for the decreased intrarenal RAS expression observed in our study. While the treated controls had no changes in plasma leptin, they showed decreased medullar ACE expression and activity with SGLT2 inhibition compared to untreated control rats suggesting a direct effect on the intrarenal RAS independent of its effect on plasma leptin.

SGLT2 inhibitors are known as anti-diabetic drugs, so we explored if EMPA would reduce fasting glucose or insulin in the PCOS model. We did not observe any changes in either of these parameters or insulin sensitivity, in agreement with studies in women with PCOS [14]. Furthermore, we observed no impact on HbA1c in PCOS after three weeks of EMPA treatment, though given that the half-life of rat red blood cells is about two months [44], longer treatment may be necessary to see a reduction in HbA1c. EMPA reduced fasting glucose only in patients who already had impaired fasting glucose, not in subjects with normal fasting glucose [45]. These findings suggested that perhaps the glucose-lowering effect of EMPA requires further disease progression to T2DM to be noticeable; however, our experimental model was one of IR instead of T2DM. Moreover, it could also be possible that hyperandrogenemia blunts the beneficial effect of SGLT2 inhibition on glucose homeostasis and that correction of hyperandrogenemia may be necessary for adequate IR management in PCOS.

We did not observe any impact on albuminuria with EMPA in the PCOS model. SGLT2 inhibitors have shown to be renoprotective in patients with T2DM. There were contradictory data about SGLT2 inhibition being renoprotective in rodent models of T2DM, with other studies showing reduced [40,46], or unchanged [27] renal injury. Our data suggested that SGLT2 inhibition was not renoprotective in women with PCOS, even though we saw a small decrease in MAP that was also reported with SGLT2 inhibition in T2DM [16,19,21]. We previously showed that renal injury in the PCOS model was associated with increases in glomerular filtration rate [25] and that androgens promoted renal injury via vasodilation of the afferent arteriole and subsequent increases in the intraglomerular capillary pressure [47]. The renoprotective effect of SGLT2i in chronic kidney diseases was via reductions in single-nephron glomerular filtration rate [48]; thereby, it was possible to speculate that the direct effect of androgens in renal hemodynamics abolish the beneficial effect of SGLT2 inhibition, although this hypothesis needs to be tested.

There are few proven therapeutic options in treating cardiovascular risk factors in women with PCOS. In a recent study in women with PCOS, the inability to lose weight was the main clinical manifestation reported by those patients [49]. In the current study, the SGLT2 inhibitor EMPA showed promise in treating the androgen-induced adiposity in PCOS. Additionally, EMPA may be able to decrease the activation of some of the RAS components in women with PCOS. EMPA is known to decrease BP and albuminuria in other disease states such as T2DM. However, both of those therapeutic benefits appear limited in the presence of hyperandrogenemia, with only a small decrease in BP and no changes in albuminuria or IR. Our study shows that SGLT2 inhibition was highly beneficial to treat some, but not all, of the cardiometabolic dysregulations in PCOS, paving the way to novel combination therapies for this pathology.

## 4. Material and Methods

### 4.1. Animals 

Three-week-old female Sprague Dawley rats were obtained from Envigo (Indianapolis, IN, USA). At four weeks of age, rats were randomly assigned to be implanted subcutaneously with continuous-release dihydrotestosterone (DHT) pellets (7.5 mg/90 days; Innovative Research of America, Sarasota, FL, USA) or sham surgery (Control) under isoflurane anesthesia, as we previously reported [25]. Rats were maintained on a standard rat chow diet (Teklad 22/5 Rodent Diet #8640; Envigo, Indianapolis, IN, USA), housed in temperature-controlled rooms with *ad libitum* food and water, and a constant light/dark cycle (12 h/12 h). Animals were followed up for 90 days. All experimental protocols were performed in accordance with the National Institutes of Health Guide for the Care and Use of Laboratory Animals, 8th Edition, 2011, and approved by the Institutional Animal Care and Use Committee of the University of Mississippi Medical Center (Protocol 1501, approved 4 May 2017).

The sodium-glucose cotransporter-2 inhibitor empagliflozin (EMPA, 10 mg/kg/day, AChemBlock, Burlingame, CA, USA) was administered in the drinking water (vehicle) at a dose shown to be effective in lowering BP and HbA1c in other rodent models [27,50]. To aid in solubility, EMPA was dissolved in drinking water and heated to 67 °C for 30 min. These solubilizing conditions were chosen as it has been reported that only 10% of EMPA is degraded after incubating EMPA in aqueous solution at 110 °C for 24 h [51]. There were three different cohorts of rats: one cohort of Control and PCOS rats (n = 6–8 per group) was used to assess the impact of hyperandrogenemia on renal glucose transporter mRNA expression; and two identically cohorts treated with EMPA were used for metabolic studies and blood pressure determinations. EMPA treatment was administrated during the last 3 weeks of the 90-day DHT treatment.

### 4.2. Food Intake, Anthropometric Measurements, and Body Composition

Food intake and body weight were recorded weekly before EMPA administration. During EMPA treatment, food intake and fluid intake were recorded daily and body weight was recorded weekly. Body composition (fat and lean mass) was measured before and after EMPA treatment by EchoMRI (4in1-900 model Body Composition Analyzer, EchoMRI, Houston, TX, USA), as we previously reported [30]. At the end of the experimental period, body length (nose–anus length) was measured to calculate BMI.

### 4.3. Metabolic Determinations 

At 16 weeks of age (3 weeks after EMPA treatment), blood from rats fasted for 6 h was obtained from clipped tails. Fasting glucose, low-density lipoprotein (LDL) cholesterol, high-density lipoprotein (HDL) cholesterol, total cholesterol, and triglycerides levels were measured using VET Axcel Chemistry Analyzer (Alfa Wassermann Diagnostic Technologies, West Calawell, NJ, USA) and reported as mg/dL. Fasting insulin was measured by a commercially available ELISA kit (Crystal Chem. Inc., Elk Grove Village, IL, USA) and reported as µU/mL. Insulin sensitivity was assessed using the quantitative insulin sensitivity check index (QUICKI) as we previously reported [29]. Then, rats were euthanized, and blood was collected for DHT, leptin, and HbA1c levels. Plasma leptin was measured by ELISA (R & D Systems, Inc., Minneapolis, MN, USA) as we previously reported [30]. Leptin was reported as ng/mL. Serum DHT was measured with commercially available DHT RIA kit (Beckman Coulter, Inc., Brea, CA, USA) as we previously reported [30]. DHT was reported as pg/mL. HbA1c was measured by a commercially available direct enzymatic HbA1c assay (Crystal Chem. Inc., Elk Grove Village, IL, USA) according to manufacturer’s recommendations. HbA1c was reported as %.

### 4.4. Urine Analysis

At 16 weeks of age (3 weeks after EMPA treatment), rats were placed individually in metabolic cages for 24-h urine collection. Urine was centrifuged at 2100× *g* for 20 min at 4 °C, aliquoted, and centrifuged again at 2100× *g* for 20 min at 4 °C. Supernatants were stored at −80 °C. Urine glucose and creatinine levels were measured using VET Axcel Chemistry Analyzer and reported as mg/dL and mg/dL, respectively. Urinary albumin was measured by commercially available ELISA kit (Nephrat ELISA, Exocell, Philadelphia, PA, USA) according to the manufacturer’s recommendations. Urine ketones were measured by Ketostix according to manufacturer’s recommendations (Ascensia Diabetes Care, Parsippany, NJ, USA).

### 4.5. Tissue Collection

At 16 weeks of age (3 weeks after EMPA treatment), rats were euthanized for renal tissue collection. Under isoflurane gas, rats were perfused (12 mL/min) with a 0.9% NaCl solution containing 2% heparin by volume via the infrarenal aorta. Immediately after completion of the perfusion, the kidneys were removed, the cortex and medulla of each kidney were separated, snap-frozen in liquid nitrogen, and stored at −80 °C.

### 4.6. mRNA Expression Quantification

Renal total RNA was extracted with TRI-Reagent (Molecular Research Center, Inc., Cincinnati, OH, USA), DNAse treated with Turbo DNA-free kit (ThermoFisher Scientific, Waltham, MA, USA), quantified, and reverse transcribed with SuperScript IV reverse transcriptase (ThermoFisher Scientific, Waltham, MA, USA) as we previously reported [30]. Gene expression was quantified by quantitative RT-PCR using TaqMan technology and Luna Universal Probe qPCR Master Mix (New England Biolabs, Ipswich, MA, USA). TaqMan Assays (ThermoFisher Scientific, Waltham, MA, USA) are reported in Table 1. PCR product quantification was performed by the relative quantification method and expressed as arbitrary units (AU) standardized against β-actin [30].

### 4.7. Protein Expression Quantification

Western blotting was performed similarly to as we previously reported [52]. Renal cortex and medulla samples were homogenized in radioimmunoprecipitation assay buffer supplemented with Halt protease and phosphatase inhibitor cocktail (ThermoFisher Scientific, Waltham, MA, USA). Total protein was quantified with bicinchoninic acid protein assay kit (ThermoFisher Scientific, Waltham, MA, USA). Fifty micrograms of total protein were separated by SDS-PAGE with 10% Criterion TGX Stain-Free Precast Gels (Bio-Rad, Hercules, CA, USA) and transferred to LF-PVDF membranes (Millipore, Burlington, MA, USA). Blotted membranes were processed and imaged for stain-free technology quantification [53]. Membranes were blocked with 5% nonfat dry milk in Tris-buffered saline containing 0.1% Tween 20 (TBST) for 1 h at room temperature. Membranes were then incubated in anti-ACE (1:10,000; Abcam, Cambridge, MA, USA, ab254222) or anti-ACE2 (1:3,000; Abcam ab108252, Cambridge, MA, USA) primary antibodies overnight at 4 °C. Then, membranes were probed with horseradish peroxidase-conjugated goat anti-rabbit secondary antibody (1:20,000; Jackson ImmunoResearch, West Grove, PA, USA, 111-035-003) for 1 h at room temperature. Detection by chemiluminescence was performed with SuperSignal West Pico PLUS (ThermoFisher Scientific, Waltham, MA, USA). Digital images were acquired with ChemiDoc MP image system (Bio-Rad, Hercules, CA, USA) and quantified with Image Lab 6 (Bio-Rad, Hercules, CA, USA). Protein expression was normalized to total protein detected by stain-free technology [53].

### 4.8. Renal ACE and ACE2 Enzymatic Activity

Kidney cortex and medulla were homogenized in ice-cold borate buffer (400 mM H_3_BO_3_; 900 mM NaCl; 340 mM sucrose; pH = 7.2) supplemented with cOmplete Mini EDTA-free protease inhibitor cocktail (Roche Diagnostics GmbH, Mannheim, Germany) at a ratio of 100 mg tissue/mL buffer. Homogenates were centrifuged at 2400× *g* for 15 min at 4 °C. The supernatant was diluted 4-fold in homogenization buffer and stored at −20 °C until activity assays were performed. Protein concentration was determined according to Bradford [54] with modifications described by Zor and Selinger [55] using bovine serum albumin as the standard.

ACE activity was determined fluorometrically [56,57,58] with some modifications as described below. Assays were carried out using 10 µL of diluted tissue homogenate in 190 µL of assay buffer (400 mM H_3_BO_3_; 300 mM NaCl; 1 µM Zn(SO_4_)*7H_2_O; pH = 8.3) supplemented with 1 mM Z-Phe-His-Leu-OH (Z-Phe, Bachem, Torrance, CA, USA). Samples were incubated for 30 min at 37 °C in a circulating water-bath. The enzymatic reaction was stopped by the addition of 1.5 mL 0.28 N NaOH. Sample blanks were prepared by adding tissue samples after the addition of NaOH. Then, 100 µL of freshly prepared o-phthaldialdehyde (150 mM in methanol; Sigma, St. Louis, MO, USA) was added and incubated for 10 min at room temperature. Later, 200 µL of 3N HCl was added, and the assay mixture was centrifuged at 1000× *g* for 5 min at room temperature. Finally, 200 µL of supernatant was transferred to a black microplate and measured fluorometrically (λex: 360nm; λem: 465nm) using a Synergy H1MF microplate reader (BioTek Instruments, Winooski, VT, USA). Serial dilutions of the di-peptide His-Leu (Bachem, Torrance, CA, USA) were used as the standard. ACE enzymatic activity was normalized by protein content and expressed as nmol/min/mg protein. The specificity of the assay was determined by using assay buffer containing 10 µM enalapril maleate salt (Sigma, St. Louis, MO, USA).

Renal cortex and medulla ACE2 activity was determined as previously reported [59,60,61,62,63] with the following modifications. The tissue homogenates were prepared as described above. Diluted tissue homogenate (2 µL) was pre-incubated for 30 min at 37 °C in the presence or absence of an ACE2 inhibitor, MLN-4760 (Millipore Sigma, St. Louis, MO, USA) (200 µM), in 48 µL of assay buffer (50 mM Tris-HCL; 250 mM NaCl; 1 µM Zn(SO_4_)*7H_2_O; 10 µM captopril; Ph = 7.0). After pre-incubation, 50 µL of 20 µM Mca-APK(Dnp) (GenScript Biotech, Piscataway, NJ, USA) was added to samples incubated with and without MLN-4760. Samples were read fluorometrically (λex: 320nm; λem: 420nm) using a microplate reader (Synergy H1MF) for 40 min at 37 °C. Serial dilutions of Mca-PL (Enzo Life Sciences, Farmingdale, NY, USA) were used as the standard. Values at 10 min of incubation were subtracted from values at 40 min of incubation. After internal normalization for each tissue sample, specific ACE2 activity was calculated by subtracting the value in the presence of MLN-4760 from the value without the ACE2 inhibitor. Tissue homogenate rates of hydrolysis were normalized by protein content, and the rates of hydrolysis were expressed as pmol/min.mg protein.

### 4.9. Blood Pressure Measurement

After 8 weeks of DHT, a cohort of PCOS and control rats (n = 8 per group), while under isoflurane gas, were implanted with radiotelemetry transmitters (HD-SD10; Data Sciences International, St. Paul, MN, USA) into the infrarenal abdominal aorta, as we previously reported [30]. After a 10-day recovery period, mean arterial pressure (MAP), systolic blood pressure (SBP), diastolic blood pressure (DBP) were monitored continuously in freely moving, conscious animals during four days of baseline measurements and three weeks of EMPA treatment. Telemetry measurements were obtained during a 10-s sampling period (500 Hz), recorded every 40 s, and daily averaged as we previously reported [30]. Light/dark cycle variations (constant light/dark cycle of 12 h) of mean arterial BP were analyzed throughout the study.

### 4.10. Statistical Analysis

All data are expressed as mean ±SEM. Data were analyzed by two or three-way analysis of variance (ANOVA) or by two-way repeated measures ANOVA followed by Tukey’s multiple comparisons *post hoc* tests. Differences were considered statistically significant at *p* < 0.05. Statistical analyses were performed with GraphPad Prism 8.1 (GraphPad, La Jolla, CA, USA).

## Figures and Tables

**Table 1:** TaqMan Assay IDs.

Gene Name	Gene Symbol	TaqMan Assay ID
Glucose Transporter-1 (GLUT1)	*Slc2a1*	Rn01417099_m1
Glucose Transporter-2 (GLUT2)	*Slc2a2*	Rn00563565_m1
Sodium-Glucose Cotransporter-1 (SGLT1)	*Slc5a1*	Rn01640634_m1
Sodium-Glucose Cotransporter-2 (SGLT2)	*Slc5a2*	Rn00574917_m1
Sodium-Glucose Cotransporter-3 (SGLT3)	*Slc5a4*	Rn01429310_m1
Sodium-Glucose Cotransporter-4 (SGLT4)	*Slc5a9*	Rn01761671_m1
Sodium-Glucose Cotransporter-5 (SGLT5)	*Slc5a10*	Rn01773089_m1
Renin	*Ren*	Rn00561847_m1
Angiotensinogen	*Agt*	Rn00593114_m1
Angiotensin-Converting Enzyme	*Ace*	Rn00561094_m1
Angiotensin-Converting Enzyme 2	*Ace2*	Rn01416293_m1
Angiotensin II Receptor Type 1a	*Agtr1a*	Rn02758772_s1
β-Actin	*Actb*	Rn00667869_m1

## Data Availability

Data is contained within the article or Supplementary Material.

## Supplementary Materials

The following are available online at https://www.mdpi.com/1422-0067/22/5/2576/s1, Figure S1: Effect of EMPA on renal ACE and ACE2 protein expression in PCOS.
