# Supplementary material for: Impact of SGLT-2 Inhibition on Cardiometabolic Abnormalities in a Rat Model of Polycystic Ovary Syndrome
Source: Int J Mol Sci. 2021 Mar 4;22(5):2576. doi: 10.3390/ijms22052576 (PMC7962009; doi:10.3390/ijms22052576)
Supplement: Supplementary file 1 [file ijms-22-02576-s001.pdf]

Supplementary Figure S1

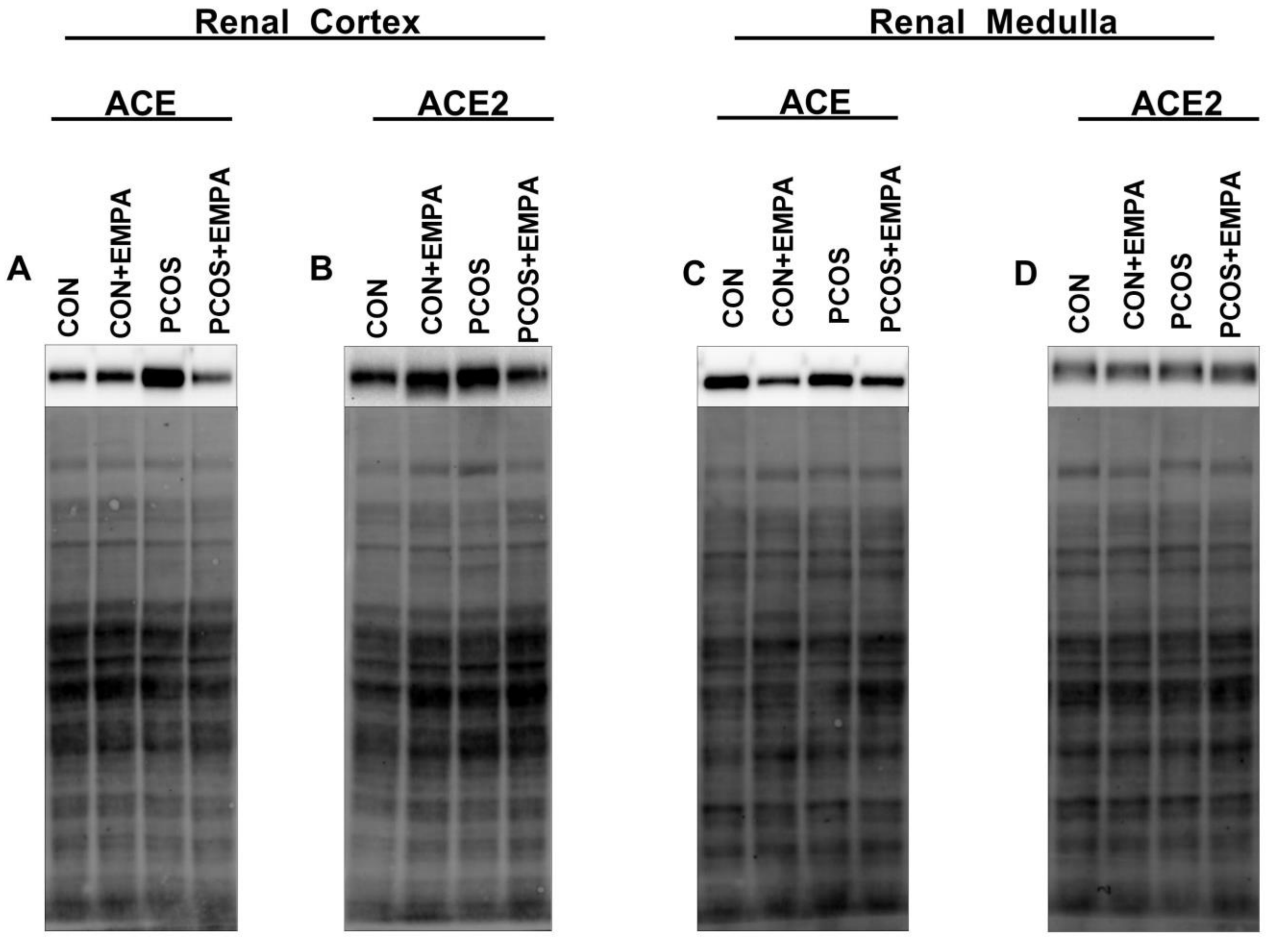

Supplementary Figure S1: Effect of EMPA on renal ACE and ACE2 protein expression in PCOS. Effect of EMPA on renal (A) cortical Angiotensin-Converting Enzyme (ACE), (B) cortical Angiotensin-Converting Enzyme 2 (ACE2), (C) medullar ACE, and (D) medullar ACE2 protein expression after 3 weeks of EMPA treatment. Top panel: ACE or ACE2. Bottom panel: Stain-Free blots used for total protein content (TPC) quantification and ACE/ACE2 expression normalization. CON: Control, CON+EMPA: Control+Empagliflozin, PCOS: Polycystic Ovary Syndrome, PCOS+EMPA: Polycystic Ovary Syndrome+Empagliflozin.
